# Supplementary material for: Comparative multiomics analysis of cell physiological state after culture in a basket bioreactor
Source: Sci Rep. 2022 Nov 23;12:20161. doi: 10.1038/s41598-022-24687-4 (PMC9686226; doi:10.1038/s41598-022-24687-4)

66040.1:107313732:107313840:-@NW_023666040.1:107305290:107305395:-@NW_023666040.1:107300120:107

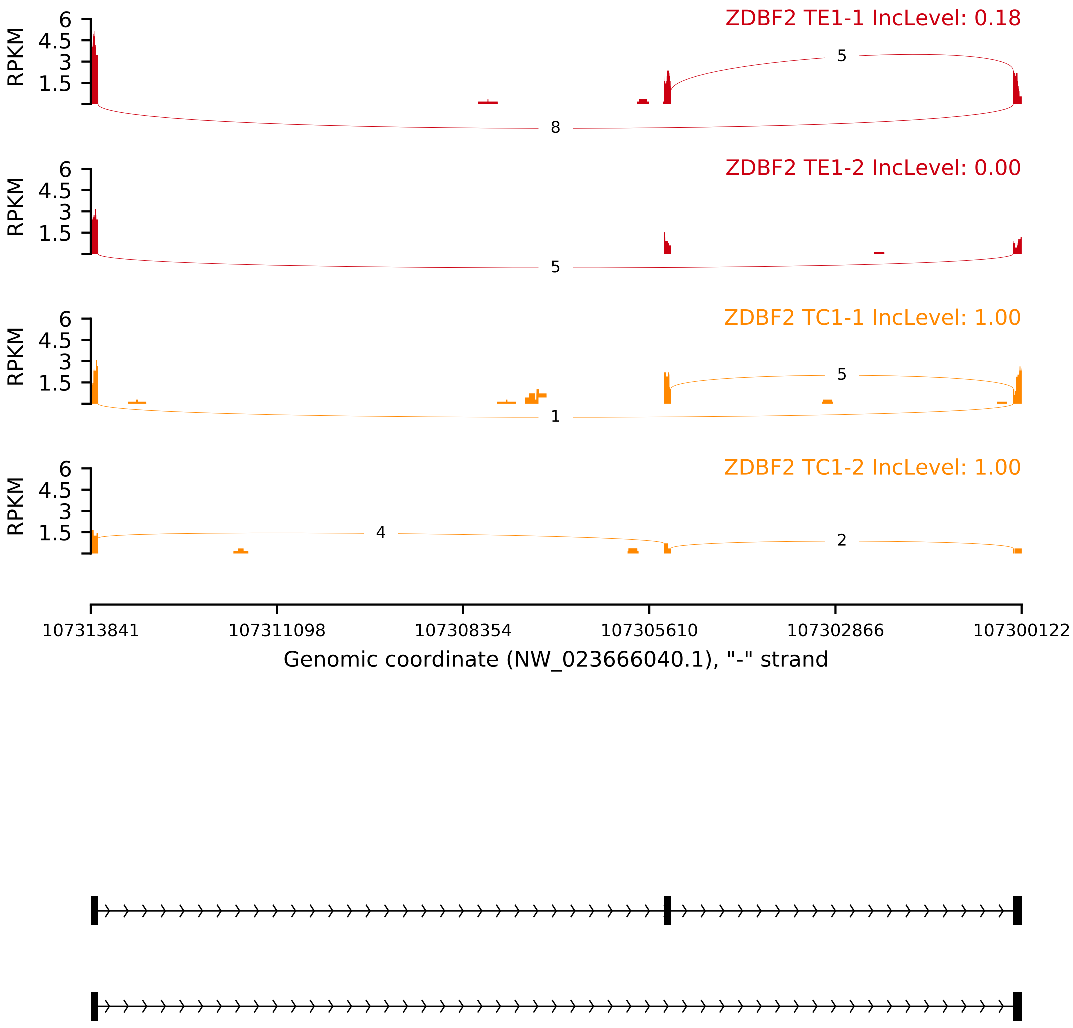

Supplement: Supplementary file 1 — Supplementary Information 1. [file 41598_2022_24687_MOESM1_ESM.zip › raw data/Transcriptomic raw data/7.AS/2.dasplot/TE1vsTC1/SE/11_ZDBF2_NW_023666040.1_107313732_107313840_-@NW_023666040.1_107305290_107305395_-@NW_023666040.1_107300120_107300247_-.pdf]
